# The prevalence of domestic violence in primary care patients in Slovenia in a five-year period (2005-2009)

**DOI:** 10.3325/cmj.2011.52.728

**Published:** 2011-12

**Authors:** Nena Kopčavar Guček, Igor Švab, Polona Selič

**Affiliations:** Department of Family Medicine, Faculty of Medicine, University of Ljubljana, Ljubljana, Slovenia

## Abstract

**Aim:**

To estimate the prevalence of exposure to domestic violence in primary care patients in Slovenia and determine the associated factors.

**Methods:**

In a systematic cross-sectional survey, 70 physicians from 70 family medicine practices from urban and rural settings conducted interviews with every fifth patient from January 15 to February 15, 2010.

**Results:**

Of 2075 patients (98.8% response rate), 372 (17.9%) were exposed to psychological or physical violence in the family in the last five years. Factors that increased the chances of exposure to psychological and physical violence were female sex (odds ratio [OR], 3.27; 95% confidence interval [CI], 2.24-4.76; *P* < 0.001; OR, 4.52; 95% CI, 2.83-7.20; *P* < 0.001, respectively) and formal divorce (OR, 2.08; 95% CI, 1.35-3.21; *P* = 0.001; OR, 2.72; 95% CI, 1.73-4.29; *P* < 0.001, respectively). Factors that decreased the chances of exposure to psychological violence were age of 65 years or above (OR, 0.56; 95% CI, 0.33-0.96, *P* = 0.035) and single status (OR, 0.43; 95% CI 0.21-0.86, *P* = 0.016), while age of 65 years or above (OR, 0.43; 95% CI, 0.23-0.79, *P* = 0.007) and parenting of two children (OR, 0.51; 95% CI, 0.29-0.90, *P* = 0.020) decreased the chances of exposure to physical violence.

**Conclusions:**

We found the rate of exposure to psychological and physical violence of 17.9%, which indicates that this problem is a serious public health issue that needs to be addressed by adequate measures. The identified risk and protective factors could serve as a valid guidance for family physicians dealing with physical violence.

Domestic violence is a serious health issue, with consequences ranging from physical impairments to psychological symptoms, physical trauma, and death (1-3). Its prevalence is between 5% and 30% (4-6), and about 90% of the perpetrators are family members (1). The exposure to violence inevitably leads to more frequent use of health services, while unrecognized causes of health problems in victims of violence can lead to unnecessary consultations, unwarranted diagnostic procedures, and ineffective health care (5-10). Health services often miss the opportunity to prevent violence (11), probably because victims hesitate to disclose it and medical health providers hesitate to ask about it, even if a number of guidelines and recommendations has been published (12-17). A meta-analysis (18) has showed that 63% of female patients in primary health care would approve of screening on domestic violence, and the percentage is even higher among those who have experienced violence (18). However, despite the recommendations of professional organizations, only 10% of physicians actively ask their patients about violence (19). The aim of the study was to estimate the prevalence of domestic violence in family care settings in Slovenia and to identify the factors influencing it.

## Methods

The study included 70 general practitioners (GP) who interviewed every fifth patient about domestic violence exposure from January 15, 2010 till either 30 patients were interviewed or February 15, 2010. The practices were selected from both urban and rural settings, serving populations with diverse socio-economic and ethnic characteristics; the diversity and geographical representativeness of family care settings followed the study design described by Svab et al (20). The participants in the systematic sample were 18 years old or older, visited their GP for health problems, and were examined for any reason. Visits for administrative purposes were excluded, and no one was accompanied by another person. The eligibility criteria were their age, purpose of visit, and willingness to participate. The short version of Domestic Violence Exposure Questionnaire introduced by Selic et al (21) (web-extra material) (web extra material 1) was administered by the GP after the examination and consultation about the health problem that was the reason for the visit. Patients were invited to participate and explained that participation was not obligatory. Of 2100 invited patients, 2075 were included in the analysis (98.8% response rate); the 25 (1.2%) people who did not want to participate did not explain their motivation.

### Procedure and measures

Using the Domestic Violence Exposure Questionnaire, derived from the work of Heise and Garcia-Moreno (22), the patients were asked about the exposure to psychological or/and physical violence, the perpetrator, and the frequency of exposure, especially in the family (ie, “In the past five years, have you ever been beaten, slapped, kicked or in any other way exposed to physical violence at home?”).

Psychological violence exposure was assessed with the question: *“*In the past five years, have you been humiliated, subjected to threats, insult or intimidation, or in any way emotionally affected within the family?” If they answered yes, they were asked the same questions as in the case of physical violence.

The Domestic Violence Exposure Questionnaire – short form was clinician-administered and consisted of 15 questions about sex, age, the number of children, marital status, the number of divorces, and place of residence, and about exposure to violence, frequency of exposure to violence, and the perpetrator of the violence (web-extra material). The study was approved by the National Medical Ethics Committee of the Republic of Slovenia.

### Statistical analyses

The sample data were presented by frequencies and percentages. Univariate comparisons were made using χ^2^ test. Multivariate binary logistic regression analysis was used to determine the risk factors for psychological and physical violence. The modeling included all the variables from the questionnaire. With regard to each predictive variable in the logistic model, the Wald χ^2^ value, statistical significance (*P* value), odds ratios (OR), and 95% confidence intervals (CI) were calculated. Statistical analysis was performed with SPSS 15.0 software (SPSS Inc., Chicago, IL, USA). *P* < 0.05 was set as the level of statistical significance.

## Results

### Demographic characteristics of participants

The sample included 2075 individuals (98.8% response rate), 768 (37.0%) men and 1307 (63.0%) women (Table 1). The majority (82.1%, n = 1703) were not exposed to psychological or physical violence in the family, including coerced sex, during the previous five years. The other 375 participants (17.9%) reported exposure to some types of domestic violence; 167 to physical violence (22 [13.2%] men and 145 [86.8%] women) and 205 to psychological violence (36 [17.6%] men and 169 [82.4%] women). Out of 167 patients exposed to physical violence, only 7 (4.2%) reported coerced sexual intercourse. There were no significant differences in sex between the groups.

**Table 1:** Sex, age, intimate relationship status, residency, and the number of children of participants

		No. (%) of participants who experienced violence:			
	All (n = 2075)	psychological (n = 205)	physical (n = 167)	P*	total (n = 372)
**Age (years):**				0.680	
up to 35	467 (22.5)	51 (24.9)	47 (28.1)		98 (26.3)
36-49	622 (30.0)	63 (30.7)	56 (33.5)		119 (32.0)
50-64	577 (27.8)	56 (27.3)	41 (24.6)		97 (26.1)
65 and above	409 (19.7)	35 (17.1)	23 (13.8)		58 (15.6)
**Sex:**				0.255	
male	768 (37.0)	36 (17.6)	22 (13.2)		58 (15.6)
female	1307 (63.0)	169 (82.4)	145 (86.8)		314 (84.4)
**Intimate partnership status:**				0.183	
living in intimate partnership	1512 (72.9)	156 (76.1)	113 (67.7)		269 (72.3)
intimate partnership ended	311 (15.0)	36 (17.6)	38 (22.8)		74 (19.9)
single	252 (12.1)	13 (6.3)	16 (9.6)		29 (7.8)
**Divorce:**					
never divorced	1862 (89.7)	170 (82.9)	132 (79.0)		302 (81.2)
formally divorced	213 (10.3)	35 (17.1)	35 (21.0)		70 (18.8)
**Number of children:**					
none	438 (21.1)	35 (17.1)	41 (24.6)		76 (20.4)
one	498 (24.0)	55 (26.8)	41 (24.6)		96 (25.8)
two	760 (36.6)	82 (40.0)	49 (29.3)		131 (35.2)
three or more	379 (18.3)	33 (16.1)	36 (21.6)		69 (18.5)
**Residency:**				0.042	
rural	694 (33.4)	63 (30.7)	57 (34.1)		120 (33.4)
suburban	388 (18.7)	33 (16.1)	41 (24.6)		74 (18.7)
urban	993 (47.9)	109 (53.2)	69 (41.3)		178 (47.9)

*χ^2^ test.

The age structure of the whole sample was as follows: up to 35 years, 22.5% (n = 467); 36-49 years, 30.0% (n = 622); 50-64 years, 27.8% (n = 577); and 65 years or above, 19.7% (n = 409), with a mean age of 49.4 ± 16.1. The victims of psychological abuse were 48.6 ± 15.6 years old, while the victims of physical violence were 46.4 ± 16.5 years old.

### Domestic violence exposure: types and perpetrators

A total of 57 (27.8%) and 60 participants (35.9%), respectively, were exposed to psychological and physical violence rarely (once or twice a year); 76 (37.1%) and 60 (35.9%), respectively, were exposed sometimes (once a month); 35 (17.1%) and 34 (20.4%), respectively, were exposed often (once or twice a week); and 32 (15.6%) and 11 (6.6%) respectively, were exposed constantly (up to twice a week). Those who experienced psychological violence were significantly more frequently exposed to it than those who experienced physical violence (*P* = 0.023) (Table 2).

**Table 2:** The frequency of physical and psychological domestic violence exposure and its perpetrators among family practice patients

	No. (%) of family practice patients who experienced		P*
	psychological violence	physical violence	
**Frequency of violence exposure:**			
rarely	57 (27.8)	60 (35.9)	0.023
sometimes	76 (37.1)	60 (35.9)	
often	35 (17.1)	34 (20.4)	
constantly	32 (15.6)	11 (6.6)	
cannot decide	5 (2.4)	2 (1.2)	
**Perpetrator:**			0.124
other family members	78 (38.0)	50 (29.9)	
partner	127 (62.0)	117 (70.1)	

*χ^2^ test.

The perpetrators of psychological violence were other family members in 38.0% (n = 78) of the cases and the partner in 62.0% (n = 127) of the cases. Similarly, the partner was involved in 70.1% (n = 117) of the cases of physical violence, and other family members in 29.9% (n = 50) of the cases (Table 2).

### Factors influencing psychological and physical violence

The modeling process explained 8% of the variance of psychological domestic violence (Nagelkerke R^2^ = 0.080; *P* < 0.001) and 13% of the variance of physical violence (Nagelkerke R^2^ = 0.131; *P* < 0.001) (Table 3)

**Table 3:** Logistic regression model of the associations between exposure to psychological and physical violence and patients` characteristics

	No. (%) of patients who did not experience violence (n = 1703)	Patients who experienced violence					
		psychological (n = 205)			physical (n = 167)		
		No. (%)	odds ratio (95% confidence interval)	*P*	No. (%)	odds ratio (95% confidence interval)	*P*
**Age (years):**							
up to 35	369 (21.7)	51 (24.9)	1.00 (reference)		47 (28.1)	1.00 (reference)	
36-49	503 (29.5)	63 (30.7)	0.72 (0.46-1.12)	0.139	56 (33.5)	0.88 (0.54-1.41)	0.585
50-64	480 (28.2)	56 (27.3)	0.68 (0.42-1.08)	0.101	41 (24.6)	0.66 (0.39-1.10)	0.112
65 and above	351 (20.6)	35 (17.1)	0.56 (0.33-0.96)	0.035	23 (13.8)	0.43 (0.23-0.79)	0.007
**Sex:**							
male	710 (41.7)	36 (17.6)	1.00 (reference)		22 (13.2)	1.00 (reference)	
female	993 (58.3)	169 (82.4)	3.27 (2.24-4.76)	<0.001	145 (86.8)	4.52 (2.83-7.20)	<0.001
Intimate partnership status:							
living in intimate partnership	1243 (73.0)	156 (76.1)	1.00 (reference)		113 (67.7)	1.00 (reference)	
intimate partnership ended	237 (13.9)	36 (17.6)	1.04 (0.67-1.62)	0.860	38 (22.8)	1.54 (0.98-2.42)	0.064
single	223 (13.1)	13 (6.3)	0.43 (0.21-0.86)	0.016	16 (9.6)	0.54 (0.28-1.06)	0.072
Divorce:							
never divorced	1560 (91.6)	170 (82.9)	1.00 (reference)		132 (79.0)	1.00 (reference)	
formally divorced	143 (8.4)	35 (17.1)	2.08 (1.35-3.21)	0.001	35 (21.0)	2.72 (1.73-4.29)	<0.001
Number of children:							
none	362 (21.3)	35 (17.1)	1.00 (reference)		41 (24.6)	1.00 (reference)	
one	402 (23.6)	55 (26.8)	1.07 (0.63-1.84)	0.798	41 (24.6)	0.65 (0.38-1.14)	0.134
two	629 (36.9)	82 (40.0)	1.07 (0.62-1.83)	0.810	49 (29.3)	0.51 (0.29-0.90)	0.020
three or more	310 (18.2)	33 (16.1)	0.92 (0.49-1.69)	0.778	36 (21.6)	0.80 (0.44-1.47)	0.470
Residency:							
rural	574 (33.7)	63 (30.7)	1.00 (reference)		57 (34.1)	1.00 (reference)	
suburban	314 (18.4)	33 (16.1)	0.89 (0.56-1.40)	0.617	41 (24.6)	1.19 (0.76-1.86)	0.446
urban	815 (47.9)	109 (53.2)	1.11 (0.79-1.57)	0.537	69 (41.3)	0.76 (0.51-1.12)	0.158

Female sex (OR, 3.27; 95% CI, 2.24-4.47) and formal divorce (OR, 2.08; 95% CI, 1.35-3.21) increased the likelihood of psychological violence exposure in patients, while age of 65 years or above (OR, 0.56; 95% CI, 0.33-0.96) and single status (OR, 0.43; 95% CI, 0.21-0.86) decreased it. Age of 65 years or above (OR, 0.43; 95% CI, 0.23-0.79) and parenting of two children (OR, 0.51; 95% CI, 0.29-0.90) decreased the likelihood of physical violence (Table 3).

## Discussion

This study found the prevalence of domestic violence in Slovenian family practice patients in the period from 2005-2009 to be 17.9%, and identified the perpetrators and the factors associated with the exposure to psychological and physical violence in primary care patients. The exposure to psychological domestic violence was more frequent than exposure to physical violence. A significantly greater percentage of victims were women, while the perpetrators were mostly victims` intimate partners. The psychological violence exposure in patients was associated with female sex and formal divorce.

Many experts in the field have concluded that the direct approach to violence screening is the most effective one (23,24). In accordance, although there is insufficient evidence to support the efficacy of domestic violence screening in health care settings, this study used the direct case finding approach since it increases the likelihood of victims` disclosure (23,25-27). The Domestic Violence Exposure Questionnaire was constructed and tested in previous studies in Slovenian primary care (21,28,29). The prevalence of exposure to domestic violence of 17.9% in primary care patients confirmed the previous results (28,29), which found a prevalence of 12.8% in 1103 patients (28). In 2007, a similar survey reported a prevalence of 12.2% in 797 primary care patients (29) and a survey from 2009 reported a prevalence of 15.3% for some types of domestic violence during the previous five years (21).

In Slovenia, before the adoption of the Law on the Prevention of Domestic Violence, the only official data on domestic violence were collected by the police; however, the police only recorded data on reported crimes. According to these records, the number of victims of domestic crime grew by 95% from 2000 to 2007. In 2007, the police dealt with more than 2700 victims of domestic violence in a country with only about two million inhabitants (21). The lack of data on the prevalence of domestic violence in the general population raised also some doubts about former findings on domestic violence exposure, therefore the present study was performed to verify the previously reported prevalence.

Our results provided sufficient evidence about domestic violence exposure in primary care patients in Slovenia, therefore future research should be focused on determining the characteristics of domestic violence victims, for which a different approach should be used (eg, in-depth interviews with trained interviewers). Other means should be developed to encourage the victims to seek help or at least to disclose victimization to health workers. Although other studies have shown that the most effective way of domestic violence detection were face-to-face interviews by a health care provider, a self-administered questionnaire or a follow-up of the interview with a later consultation could also be used (30). This study identified some risk factors for domestic violence that help in defining the victims’ profile, although a more detailed list could be made. Previous studies showed that having experienced violence in one’s primary family increased the risk of becoming a victim or perpetrator (22). However, the present study did not investigate the family history of domestic violence. Rather the patients were asked whether they had experienced violence in the previous five years, while other similar studies only inquired about the past year or the present situation (18,23). The lifetime prevalence of violence against women is between 25% and 30%, while the annual prevalence is 2%-12% (31). The results vary depending on the screening method, the instruments used, and the health care setting (18,23,24). Our results fall within this interval.

Although the elderly have been considered as a risk group for victimization (11), this study did not confirm such findings. It found female sex and divorce to be risk factors for both types of domestic violence exposure, which is concordant with other studies (29,31). The fact that the elderly have been less at risk than the younger population may be due to lifestyle changes in the last decades. Nowadays, young people stay single and socially and financially dependent on their parents for a longer time, as opposed to the past when several generations of whole families lived together. Besides, with the increase in life expectancy, the effectiveness of diagnostic and therapeutic interventions, and quality of life, individuals above 65 years may be less vulnerable to violence.

In comparison to a study on a representative sample of Slovenian family clinic attendees (20), our study included more women, with slightly lower mean age. The predominance of women may have affected the distribution of violence according to sex in the sample.

It is also interesting to note that, in spite of a structured interview procedure, only 7 cases of coerced sexual intercourse were identified. This may be due to physicians’ lack of training or motivation, patient-physician interaction, or patients’ shame. Since the rates of sexual abuse in the majority of other studies ranged from 3.9 to 8.3% (31), this rate is extremely low and is almost certainly a false result. Therefore, a limitation of this survey is missing data on sexual violence, as we were only able to present the data on the prevalence of physical and psychological violence.

The detected frequency of violence is similar to other published data (18,23,24,31). After we have assessed the problem’s dimensions, it is necessary to implement an intervention program for the victims and a project providing support to the GPs. We believe that an exposure rate of 15% or more should be considered a serious public health issue. Further research is needed to confirm the results of this study.
