# Supplementary material for: The prevalence of domestic violence in primary care patients in Slovenia in a five-year period (2005-2009)
Source: Croat Med J. 2011 Dec;52(6):728–34. doi: 10.3325/cmj.2011.52.728 (PMC3243325; doi:10.3325/cmj.2011.52.728)
Supplement: Supplementary Table 1 [file CroatMedJ_52_s002.pdf]

Domestic Violence Exposure in Primary Care Patients Summary Sheet

1. Gender: Male Female
2. Age________ years

3. Marital status
   - ☐ in partnership (married or living with partner)
   - ☐ single
   - ☐ divorced
4. Number of children ________
5. If divorced, number of divorces ____

6. Residency / Locality
   - ☐ Rural
   - ☐ Suburban
   - ☐ Urban

7. In the past five years, have you ever been beaten, slapped, kicked or in any other way exposed to physical violence at home?

YES/NO

8. If YES: by whom?
9. If YES: How often? Tick below:

☐ rarely (up to twice a year
☐ occasionally (up to once a month)
☐ often (up to once a week)
☐ constantly (more than once a week)

10. Have you been in the last 5 (five) years, forced into sexual intercourse or any unwanted sexual behaviour?

YES/NO

11. If YES: by whom?
12. If YES: How often? Tick below:

☐ rarely (up to twice a year
☐ occasionally (up to once a month)
☐ often (up to once a week)
☐ constantly (more than once a week)

13. In the past five years, have you been humiliated, subjected to threats, insult or intimidation, or in any way emotionally affected within the family?

YES/NO

14. If YES: by whom?
15. If YES: How often? Mark below

☐ rarely (up to twice a year
☐ occasionally (up to once a month)
☐ often (up to once a week)
☐ constantly (more than once a week)

IZPOSTAVLJENOST NASILJU V DRUŽINI - ZBIRNI LIST

1. Spol M Ž
2. Starost ________ let

3. Stan
   - □ živi s partnerjem/partnerko (poročen ali v zunaj-zakonski skupnosti)
   - □ samski
   - □ ločen
4. Število otrok ________
5. Če je oseba razvezan/a, navedite število razvez ____

6. Bivališče
   - □ vaško
   - □ primestno
   - □ mestno

7. Ali ste bili v zadnjih 5 (petih) letih doma (v družini) tepeni, oklofutani, obrcani ali kakorkoli izpostavljeni telesnemu nasilju? DA NE

8. Če DA: S strani koga?
9. Če DA: Kako pogosto? označite s križcem
   - □ Zelo redko (do 2 – krat letno)
   - □ Včasih (do 1 – krat mesečno)
   - □ Pogosto (do 1 – krat tedensko)
   - □ Neprestano (več kot enkrat tedensko)

10. Ali ste bili v zadnjih 5 (petih) letih prisiljeni v spolno občevanje ali kakršnokoli neželeno spolno vedenje? DA NE

11. Če DA: S strani koga?
12. Če DA: Kako pogosto? označite s križcem
    - □ Zelo redko (do 2 – krat letno)
    - □ Včasih (do 1 – krat mesečno)
    - □ Pogosto (do 1 – krat tedensko)
    - □ Neprestano (več kot enkrat tedensko)

13. Ali ste bili v zadnjih 5 (petih) letih doma (v družini) ponižani, izpostavljeni grožnjam, žalitvam, zastraševanju ali kakorkoli čustveno prizadeti? DA NE

14. Če DA: S strani koga?
15. Če DA: Kako pogosto? označite s križcem

□ Zelo redko (do 2 – krat letno)
□ Včasih (do 1 – krat mesečno)
□ Pogosto (do 1 – krat tedensko)
□ Neprestano (več kot enkrat tedensko)
